# Nanocomposites Derived from Polymers and Inorganic Nanoparticles

**DOI:** 10.3390/ma3063654

**Published:** 2010-06-14

**Authors:** In-Yup Jeon, Jong-Beom Baek

**Affiliations:** Interdisciplinary School of Green Engineering, Ulsan National Institute of Science and Technology (UNIST), 100, Banyeon, Ulsan, 689-798, Korea

**Keywords:** nanocomposites, polymers, inorganic nanoparticles

## Abstract

Polymers are considered to be good hosting matrices for composite materials because they can easily be tailored to yield a variety of bulk physical properties. Moreover, organic polymers generally have long-term stability and good processability. Inorganic nanoparticles possess outstanding optical, catalytic, electronic and magnetic properties, which are significantly different their bulk states. By combining the attractive functionalities of both components, nanocomposites derived from organic polymers and inorganic nanoparticles are expected to display synergistically improved properties. The potential applications of the resultant nanocomposites are various, e.g. automotive, aerospace, opto-electronics, *etc.* Here, we review recent progress in polymer-based inorganic nanoparticle composites.

## 1. Introduction

Nanocomposites are as multiphase materials, where one of the phases has nanoscale additives [1]. They are expected to display unusual properties emerging from the combination of each component. According to their matrix materials, nanocomposites can be classified as ceramic matrix nanocomposites (CMNC), metal matrix nanocomposites (MMNC), and polymer matrix nanocomposites (PMNC). In this review, the recent progress in PMNC is reported.

Polymers are now the most widely used in the field of technical textiles. The widespread use of common organic polymers such as polyolefins, nylons, polyesters and polyurethanes emanates from key features such as lightweight, easy fabrication, exceptional processability, durability and relatively low cost [2,3]. A major challenge in polymer science is to broaden the application window of such materials by retaining the above features while enhancing particular characteristics such as modulus, strength, fire performance and heat resistance [3]. However, polymers have relatively poor mechanical, thermal, and electrical properties as compared to metals and ceramics. Many types of polymers such as homopolymers, co-polymers, blended polymers and modified polymers are not sufficient enough to compensate various properties, which we have demanded. Alternative approaches to improve their properties are to reinforce polymers with inclusion of fiber, whisker, platelets, or particles.

The choice of the polymers is usually guided mainly by their mechanical, thermal, electrical, optical and magnetic behaviors. However, other properties such as hydrophobic/hydrophilic balance, chemical stability, bio-compatibility, opto-electronic properties and chemical functionalities (*i.e.*, solvation, wettability, templating effect, *etc.*) have to be considered in the choice of the polymers. The polymers in many cases can also allow easier shaping and better processing of the composite materials.

The inorganic particles not only provide mechanical and thermal stability, but also new functionalities that depend on the chemical nature, the structure, the size, and crystallinity of the inorganic nanoparticles (silica, transition metal oxides, metallic phosphates, nanoclays, nanometals and metal chalcogenides). Indeed, the inorganic particles can implement or improve mechanical, thermal, electronic, magnetic and redox properties, density, refractive index, *etc.* [4].

Organic polymer-based inorganic nanoparticle composites have attracted increasing attention because of their unique properties emerging from the combination of organic and inorganic hybrid materials. Generally, the resultant nanocomposites display enhanced optical, mechanical, magnetic and optoelectronic properties. Therefore, the composites have been widely used in the various fields such as military equipments, safety, protective garments, automotive, aerospace, electronics and optical devices. However, these application areas continuously demand additional properties and functions such as high mechanical properties, flame retardation, chemical resistance, UV resistance, electrical conductivity, environmental stability, water repellency, magnetic field resistance, radar absorption, *etc.* Moreover, the effective properties of the composites are dependent upon the properties of constituents, the volume fraction of components, shape and arrangement of inclusions and interfacial interaction between matrix and inclusion. With the recent development in the nanoscience and nanotechnology fields, the correlation of material properties with filler size has become a focal point of significant interest [2].

## 2. Inorganic Nanoparticles

Compared to conventional micron-sized particles, nanoparticles have a much higher surface-to-volume ratio. As the particle size decreases, the percentage of molecules/atoms present on the surface is tremendously increased [5,6]. As a result, interparticle forces such as van der Waals and electrostatic forces, as well as magnetic attraction, become stronger. Without proper chemical treatment to reduce the surface energy, it is very common for nanoparticles to form clusters or agglomerates, which are challenging to disperse individually and uniformly in the polymer matrix, thus resulting in opaque nanocomposites akin to conventional composites [7].

**Figure 1:**
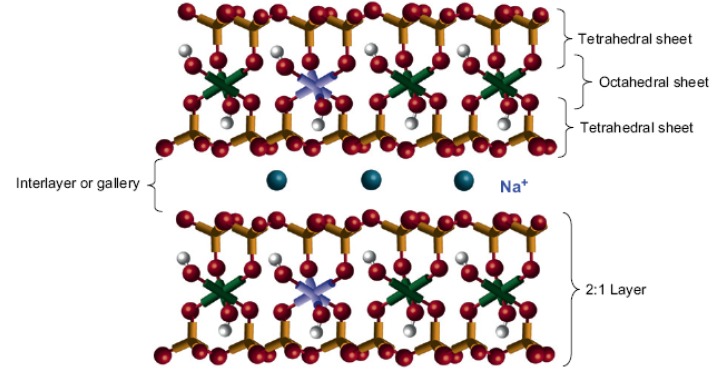
Structure of sodium montmorillonite (adapted from [11]).

The properties of the nanocomposites are contributed to the properties of the components, shape and volume fraction of the filler, the morphology of the system and the nature of the interphase that sometimes develop at the interface of the two components [8]. The extent of property enhancement depends on many factors including the aspect ratio (length/diameter) of the filler, its degree of dispersion and orientation in the matrix, and the adhesion at the filler-matrix interface [9]. For example, clays have sandwich types of structures with one octahedral Al sheet and two tetrahedral Si sheets, so-called philo-silicate. There are many types of philo-silicates: kaolinite, montmorillonite (MMT), hectrite, saponite, synthetic mica, *etc.* The MMT consists of stacked silicate sheets with lengths of about 218 nm and 1,230 nm for synthetic mica. It has the same sheet thickness of 1 nm (Figure 1) [10,11].

Since the clay particles have extremely high specific surface area (750 m^2^/g), good dispersion even at low clay contents provides an enormous amount of interfacial area [12].The unique nanometer-size of the layered silicates has characteristics such as high aspect ratios, high surface areas, and high strengths [13]. Zinc sulfide (ZnS) has excellent physical properties such as wide bandgap energy of 37 eV at 300 K [14], high refractive index (*n* = 2.36 at 620 nm) and low absorption coefficient over a broad wavelength range [15,16].

The idea is to incorporate chemically treated spherical nanoparticles with proper size, to attribute into the polymer matrix and disperse them at the nanoscale to minimize light scattering and thus, to attain high transmittance while realizing retention or improvements in some of the material and optical properties [7]. One method to facilitate dispersion of nanoparticles is to coat the nanoparticle with a thin layer of polymer to introduce steric stabilization [17]. By coating the nanoparticle with a thin layer of polymer [7,18] and surface functionalization of the nanoparticles [10,19,20,21], the van der Waals influence from the nanoparticles can be masked and the compatibility between the hosting organic polymer and inorganic nanoparticle can be improved, thereby facilitating better nanoparticle dispersion and increased loading amount.

Table 1 shows synthesis of inorganic nanoparticles from aqueous solutions and Table 2 from nonaqueous solutions [22].

**Table 1:** Inorganic nanoparticles precipitated from aqueous solutions (adapted from [22]).

Metal	Starting Material	Reducing Agent	Stabilizer ^a^	Notes	Avg Diam (nm)
Co	Co(OAc)_2_	N_2_H_4_·H_2_O	none		~20
Ni	NiCl_2_	N_2_H_4_·H_2_O+NaOH	CTAB	reaction performed at 60 °C	10-36
Ni	Ni(OAc)_2_	N_2_H_4_·H_2_O+NaOH	none		(10-20) × (200-300) rods
Cu	CuSO_4_	N_2_H_4_·H_2_O	SDS		~35
Ag	AgNO_3_	Ascorbic acid	Daxad 19		15-26
Ag	AgNO_3_	NaBH_4_	TADDD		3-5
Pt	H2PtCl_6_	potassium bitartrate	TDPC	60 °C	<1.5
Au	HAuCl_4_	trisodium citrate	S3MP	simultaneous addition of reductant and stabilizer	not stated

^a^ CTAB = cetyltrimethylammonium bromide (see section 4); SDS = sodium dedecyl sulfate; Daxad 19 = sodium salt of high-molecular-weight naphatalene sulfonate formaldegyde condensate; TADDD = bis(11-trimethylammoniumdecanoylaminoethyl)-disufide dibromide; TDPC = 3,3’-triodipropinoic acide; S3MP = sodium 3-mercaptopropionate.

**Table 2:** Inorganic nanoparticles precipitated by reduction from nonaqueous solutions (adapted from [22]).

Compd.	Starting Material	Solvent ^a^	Reductant ^b^	Stabilizer ^c^	Conditions	Product Size ^d^ (nm)
Fe	Fe(OEt)_2_	THF	NaBEt_3_H	THF	16 h at 67 °C	10-100
Fe	Fe(acac)_3_	THF	Mg^+^	THF		~8 ^e^
Fe_20_Ni_80_	Fe(OAc)_2_ Ni(OAc)_2_	EG	EG	EG	reflux (150-160 °C)	6 (A)
Co	Co(OH)_2_	THF	NaBEt_3_H	THF	2h at 23 °C	10-100
Co	CoCl_2_	THF	Mg+	THF		~12
Co_20_Ni_80_	Co(OAc)_2_ Ni(OAc)_2_	EG	EG	EG	reflux (150-160 °C)	18-22 (A)
Ni	Ni(acac)_2_	HDA	NaBH_4_	HDA	160 °C	3.7 (C)
Ni	NiCl_2_	THF	Mg^+^	THF		~94 ^e^
Ni	Ni(OAc)_2_	EG	EG	EG	reflux (150-160 °C)	25 (A)
Ru	RuCl_3_	1,2-PD	1,2-PD	Na(OAc) and DT	170 °C	1-6 (C)
Ag	AgNo_3_	methanol	NaBH_4_	MSA	room temp	1-6 (C)
Ag	AgClO_4_	DMF	DMF	3-APTMS	20-156 °C	7-20 (C)
Au	AuCl_3_	THF	K^+^(15C)_2_K^-^	THF	-50 °C	6-11 (C)
Au	HAuCl_3_	formamide	formamide	PVP	30 °C	30 (C)

*^a^* EG = ethylene glycol; DMF = dimethylformamide; HAD = hexadecylamine; THF = tetrahydrofuran; 1,2-PD = 1,2-propanediol. *^b^* See text for descriptions of reducing agents. *^c^* MSA = mercaptosuccinic acid; 3-APTMS = 3-(aminopropyl)trimethoxysilan; PVP = poly(vinylpyrrodinone); DT = dodecanethiol. *^d^* (A) = aggometrated; (C) = colloidal/monodispersed *^e^*Estimated from BET surface area assuming spherical shape.

## 3. Nanocomposite Synthesis

**Figure 2:**
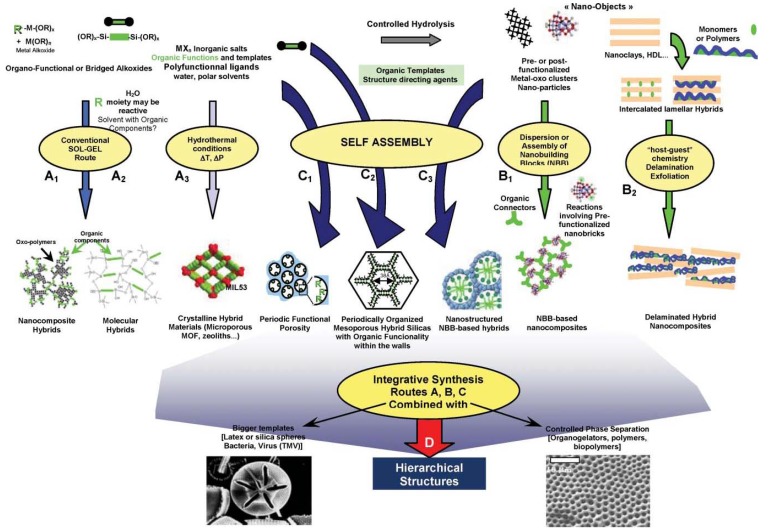
Schematic representation of the main chemical routes for the synthesis of polymer-inorganic nancomposites. Path A: sol-gel process; Path B: assembly or dispersion; Path C: self-assembly procedures; Path D: integrative synthesis. (adapted from [4]).

Many methods have been reported for the preparation of polymer-based inorganic nanoparticles composites. The important ones are: (i) intercalation of nanoparticles with the polymer or pre-polymer from solution [23,24,25]; (ii) *in situ* intercalative polymerization [26,27,28]; (iii) melt intercalation [29,30]; (iv) direct mixture of polymer and particulates [31,32,33]; (v) template synthesis [34,35]; (vi) *in situ* polymerization [36,37,38]; (vii) sol-gel process [39,40,41] (Figure 2). Publications dealing with various methods for the incorporation of nanoparticles into conducting polymers are also available. The most prominent one is probably the incorporation of inorganic nanoparticles in polymers.

Polymer based nanocomposite synthesis was effected by molecular weight, inorganic particles size and content, properties of inorganic particles.

Maleic anhydride (MA) grafted polyethylene/clay nanocomposites were prepared by simple melt compounding [42]. The exfoliation and intercalation behaviors depended on the hydrophilicity of polyethylene grafted with maleic anhydride and the chain length of organic modifier in the clay. When polyethylene has a higher grafting level of MA than the critical grafting level of MA (0.1 wt %) and the number of methylene groups in alkylamine chain has more than 16, polyethylene/clay nanocomposites are completely exfoliated.

The nano-SiO_2_ particle/linear low-density polyethylene (LLDPE) was prepared via *in situ* polymerization [43]. Only slight increase in MW (molecular weight) with the larger particles was evident. It can be observed that increased amounts of SiO_2_ resulted in decreased MW. In addition, a slightly broad MWD (molecular weight distribution) was seen in all samples. It was found that the larger particles exhibited higher activity due to fewer interactions between SiO_2_ and MAO (methylaluminoxane).

The low molecular weight nylon 6/MMT nanocomposites had regions of intercalated and exfoliated clay platelets, while the medium and high molecular weight nylon 6/MMT nanocomposites revealed well exfoliated structures. As the molecular weight increased, so did the extent of clay platelet exfoliation for the nanocomposites [44,45].

PMMA (poly(methyl methacrylate))/clay nanocomposites were synthesized via a novel pseudo-dispersion polymerization in scCO_2_ (supercritical carbon dioxide) [46]. The fluorinated surfactant-modified clay (10F-clay) can indeed serve as an effective stabilizer for PMMA polymerization in CO_2_ and help improve polymer yields compared with conventional hydrocarbon surfactant-modified clay. PMMA/layered silicate intercalated nanocomposites were synthesized using scCO_2_ [47]. At clay concentrations approaching 40 wt%, the silicate morphology is homogeneous, because the intercalated silicate structure is thermodynamically limited with regard to clay separation.

**Figure 3:**
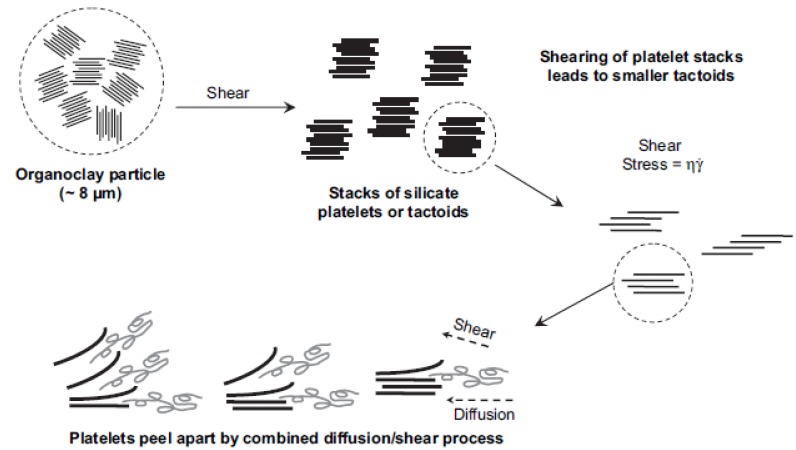
Mechanism of organoclay dispersion and exfoliation during melt processing [44].

## 4. Properties of Polymer-based Nanocomposites

Polymer-based nanoparticle nanocomposites were prepared via various processes and showed improved mechanical, thermal and electrical properties. However, the aforementioned properties of resultant nanocomposites were not always improved. For example, if one property changed for the better, another property changed for the worse. When nanocomposites are designed, one needs to take this tendency into account and find the optimum properties for specific applications.

### 4.1. Mechanical properties

Generally, the reason for adding inorganic particles into polymers is to improve its mechanical properties such as the tensile strength, modulus or stiffness via reinforcement mechanisms described by theories for nanocomposites [13,48,49,50,51]. However, poor compatibility between the polymer matrices and the inorganic particles in nanocomposites prepared by simple physical mixing will create inherent defects which, consequently, result in a deleterious effect on the mechanical properties of the nanocomposites [20,52,53].

**Figure 4:**
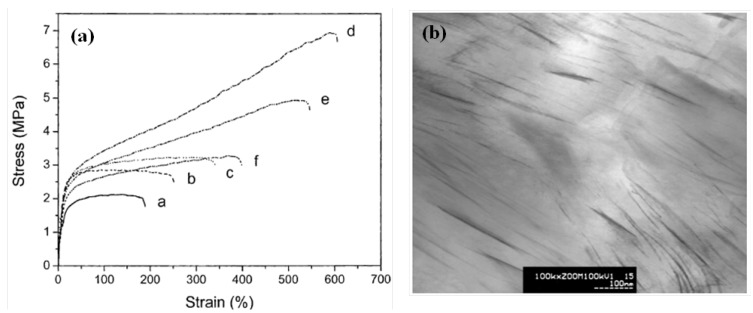
(a) Stress-strain curves for the PU/clay films with different clay contents and tensile properties: (a) 0, (b) 1, (c) 2, (d) 3, (e) 4 and (f) 5 wt %; (b) TEM microphotograph of the PU/clay nanocomposite with 5 wt % clay (adapted from [50]).

Pattanayak *et al.* prepared polyurethane (PU)-based clay nanocomposites [48]. When clay particles were fully exfoliated, modulus, tensile strength, tear strength and fracture toughness of PU/clay nanocomposites were increased by 110%, 170%, 110%, 120%, 40%, respectively, as compared with pristine PU. Note that such improvements can be attributed to clay-polymer tethering as well as hydrogen bonding between clay particles and the polymer. Also, Lee *et al.* reported that tensile strength and elongation at break of PU/clay nanocomposites increased with increasing clay content in the range of 1–3 wt %, but when the clay content is higher than 3 wt %, the tensile properties of the nanocomposites decreased slightly (Figure 4a) [50]. A transmission electron micrograph (TEM) of the cross section of a WPU/clay nanocomposite with 5 wt % clay is shown in Figure 4b. This is probably due to some degree of aggregation of the exfoliated clay platelets above the critical content.

**Figure 5:**
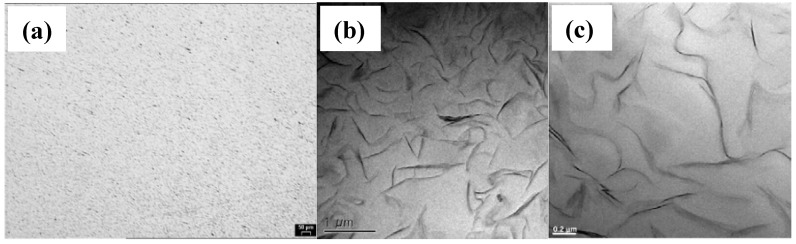
Micrographs of Epoxy/S-clay 2.5: (a) Optical micrographs of polished surface; (b) and (c) TEM micrographs (adapted from [38]).

Wang *et al.* developed a “slurry-compounding” process for the preparation of epoxy/clay nanocomposites, showing that clay uniformly dispersed and oriented randomly in the epoxy matrix (Figure 5) [13]. Both Young’s modulus and the fracture toughness are improved with the incorporation of clay and the sample containing 2.5 wt % of clay shows the highest fracture toughness.

Sarwar *et al.* reported poly(trimethylhexamethylene terephthalamide)/SiO_2_ nanocomposties prepared by a sol-gel process [54]. The values of yield stress and modulus for the composites increased with an increase in the silica content up to 10 wt % and then decreased with higher concentrations of inorganic phase.

Lu *et al.* made polyimide (PI)/ZnS nanocomposites, which showed that both the modulus and the tensile strength increased but the elongation at break decreased as ZnS load increased [55]. This can be explained by the better interfacial interaction between polymer and inorganic nanoparticles with higher surface area.

Reddy *et al.* manufactured polypropylene (PP)/nano-silica (NS) nanocomposites [56]. The modulus of PP-NS was much higher than that of pure PP, and that of PP-ENS (epoxy-resin-grafted nano-silica) was higher than that of pure PP, but lower than PP-NS. The tensile strength and elongation at break decreased for PP-NS because of NS aggregation and a lack of interfacial adhesion between the polymer and the filler. However, those of PP-ENS were increased in comparison with PP and PP-NS because of obstruction of the formation of agglomerates by epoxy-resin grafting.

The mechanical properties of nanocomposites, prepared from various polymers and inorganic particles, did not always increase. In some cases, the properties of nanocomposites were decreased by the addition of inorganic particles because of aggregation in polymer matrices. To solve this problem, the load amounts of inorganic particles were optimized or were functionalized with organic material. For example, the tensile strength and elongation at break of poly(ethylene phthalate) (PET)/nano-TiO_2_ fiber was slightly decreased as compared to that of controlled PET fiber. It was assumed that the addition of nano-TiO_2_ resulted in decreased interaction between the PET macromolecules [20]. Also, the introduction of ZnO nanoparticles into polystyrene (PS) decreased both the tensile strength and elongation to break. This implies that the interfacial adhesion is not strong enough to stand up to large mechanical forces [53], likely because the homogeneous dispersion of nanoparticles was difficult. Nano-sized particles have high surface energy and thus, they are easy to aggregate.

### 4.2. Thermal properties

For structural applications at elevated temperatures, the dimensional stability of low thermal expansion coefficient of these nanocomposites is also very important. The high thermal expansion coefficient of neat polymers causes dimensional changes during the molding process. The changes are either undesirable or, in some cases, unacceptable for certain applications.

Yu *et al.* showed that PS and aluminum nitride nanocomposite (AlN) were mixed at room temperature and then hot pressed [57]. The thermal conductivity of nanocomposites increased with increasing filler contents. The thermal diffusivity of composites decreases slightly with increasing temperature in the testing range.

**Figure 6:**
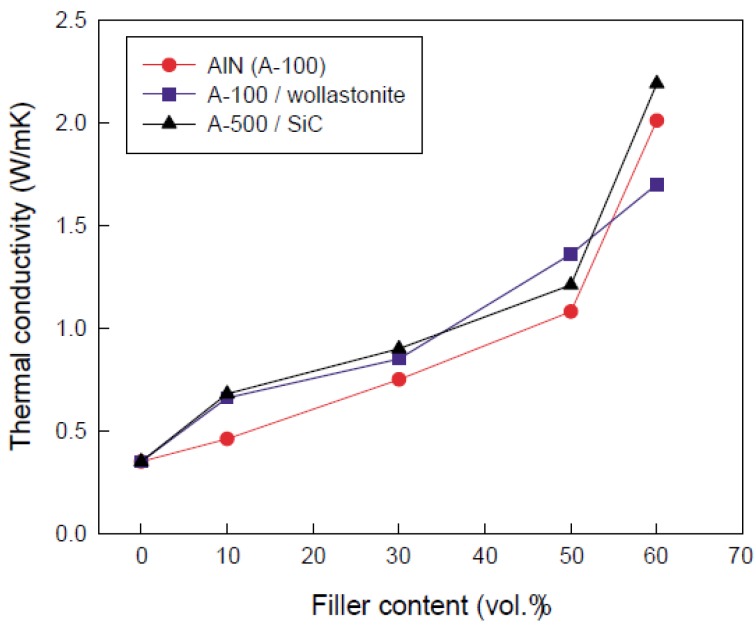
Thermal conductivity of composites containing hybrid filler (adapted from [58]).

Lee *et al.* reported that HDPE/filler nanocomposites were prepared using a mixer and fillers were used such as Wollastonite, SiC, and BN [58]. The thermal conductivity of HDPE nanocomposites was increased with increasing filler content (Figure 6).

Wu *et al.* reported that PS-encapsulated SiO_2_ nanoparticles can accelerate the crystallization of PET compared to both un-modified SiO_2_ and modified SiO_2_ [18].The PET-SiO_2_/PS nanocomposite films with 2 wt % load of PS-encapsulated SiO_2_ nanoparticles possess the fastest crystallization rate. These results reveal the nucleation effect of PS-encapsulated SiO_2_ nanoparticles in the PET matrix.

Wang *et al.* showed that PMMA/SiO_2_ and PMMA/ZrO_2_ nanocomposites were prepared using a novel nonhydrolytic sol-gel process [59]. The temperatures, where the maximum weight loss was observed by thermal decomposition of PMMA segments, all increased dramatically. The thermal decomposition temperatures of PMMA/SiO_2_ and PMMA/ZrO_2_ were profoundly improved, because network structure between inorganic and organic components reduces the movement of polymer chains, and inorganic components may retard the attack of the free radicals.

Du *et al.* prepared nylon 6/MgAl-layered double hydroxide (LDH) nanocomposites via organic modification and melt intercalation [60]. The exothermic peak temperature for pure nylon 6 is increased up to about 14 °C. Also, they prepared LLDPE/MgAl-LDH nanocomposites via melt mixing [61]. When 5 wt % of MgAl(H-DS) (organic modified MgAl-LDH) is added, exfoliated LDH layers increased endothermic peak temperature by about 5 °C. Inorganic particle layers act as nucleating agents, which have a heterogeneous nucleation effect on the crystallization temperature of polymer.

Lu *et al.* reported that silane-grafted-polyethylene/OMT nanocomposites (VTMS-g-PE/OMT) were prepared by reactive extrusion from linear low density polyethylene (LLDPE), vinyltrimethoxysilane (VTMS), organically modified montmorillonite (OMT) and dicumyl peroxide (DCP) [62]. VTMS-g-PE/OMT showed higher thermooxidative stability than that of pure LLDPE and VTMS-g-PE. The thermooxidative degradation temperature of VTMS-g-PE/OMT for a mass loss of 10% and 50% is about 23 and 16 °C, respectively, higher than that of pure LLDPE. This is because the chemically bonded silicate layers with high aspect ratios can sustain high temperatures and efficiently hinder the heat and mass transfer.

**Figure 7:**
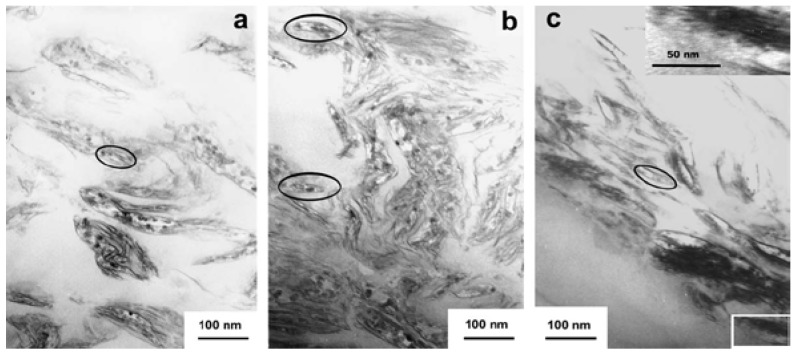
TEM of PS/LDH nanocomposites sample: (a) 5 wt %; (b) 10 wt %; (c) 20 wt %. The inset in (c) shows an enlarged image of the part marked by a rectangle (adapted from Ref. [63]).

Qiu *et al.* reported that exfoliated PS/ZnAl layered double hydroxide (PS/ZnAl-LDH) nanocomposites were synthesized by a solution intercalation method [63]. When 50% weight loss was selected as a point of comparison, the thermal decomposition temperature of exfoliated PS/ZnAl-LDH nanocomposites with 10 wt % ZnAl-LDH is 39 °C higher than that of pure PS. The thermal stability of the exfoliated nanocomposites is generally better than that of intercalated composites. TEM images with the different contents of ZnAl (DS) in the PS/LDH nanocomposites sample are shown in Figure 7. The inset in Figure 7c shows an enlarged image of the part marked by a rectangle.

Gyoo *et al.* prepared nylon 66/clay nanocomposites by melt compounding [31]. *T*_g_ and *T*_m_ of nanocomposites showed little change with increased clay content. So, crystallization temperature of nanocomposites was generally increased by about 15 °C relative to that of neat nylon 66. The crystallinity of the nanocomposites was also increased. However, at high concentrations of clay, the rate of crystallization was retarded and reduced the crystallinity.

**Figure 8:**
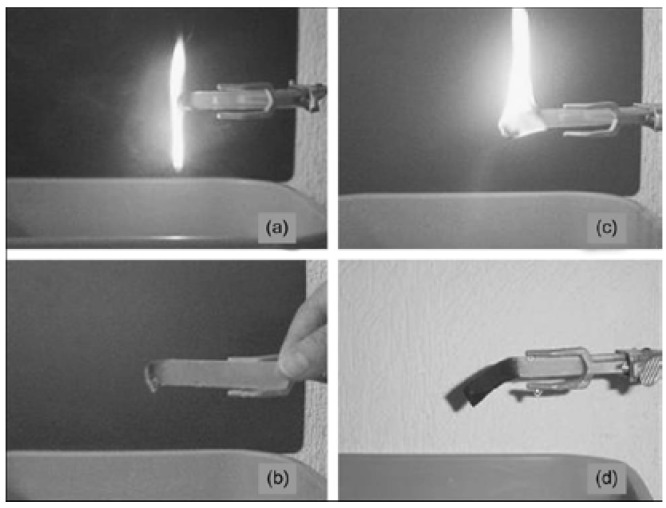
Flammability tests of: (a) EVA and (c) EVA/C20A (after 1 min of burning); (b) EVA and (d) EVA/C20A (after flame extinction). The hybrid contains 6 wt % organoclay (adapted from Ref. [64]).

Valera-Zaragoza *et al.* reported the heterophasic polypropylene(ethylenepropylene) copolymer (PP-EP)/poly(ethylene vinyl acetate) (EVA)/MMT nanocomposites [64]. Thermal degradation of the aliphatic chains in the PP-EP/EVA matrix can be retarded by an improvement in the dispersion and exfoliation of the silicate layers, which work as a barrier for heat diffusion. Organoclay concentration up to 6 wt % proportional to the retardation of thermal decomposition of nanocomposites (Figure 8).

### 4.3. Electrical properties

Nanocomposites are closely related to the design of advanced devices for electronic and optoelectronic applications. The dimensional scale for electronic devices has entered the nano-range. The utility of polymer/inorganic particle nanocomposites in these areas is quite varied involving many potential applications as well as types of nanocomposites.

Su *et al.* carried out *in situ* polymerization of polyaniline (PANi) in the presence of TiO_2_ to synthesize PANi/TiO_2_ nanocomposites [65]. The resultant nanocomposite films showed appreciable conductivity (1–10 S/cm), which was further increased after thermal treatment 80 °C for 1 h. Mo *et al.* also prepared PANi/TiO_2_ nanocomposites with TiO_2_ nanoparticles and colloids, respectively [66]. As the content of TiO_2_ increased, the dielectric constant and loss were also increased. The conductivity of nanocomposites was gradually increased as the amount of TiO_2_ increased from 1 to 5 wt %.

Olad *et al.* synthesized PANi/organophilic montmorillonite (O-MMT) and PANi/hydrophilic montmorillonite (Na-MMT) nanocomposites via *in situ* polymerization [67]. When MMT content was 5 wt %, conductivity of PANi/Na-MMT (1.201 S/cm) was lower than that of pure PANi (1.275 S/cm). However, the conductivity of PANi/O-MMT (1.650 S/cm) was slightly improved compared to that of pure PANi.

Tang *et al.* reported PPy/SiO_2_ nanocomposites prepared by *in situ* oxidative polymerization [68]. The PPy/SiO_2_ nanocomposites showed an electrical conductivity of 32.41 S/cm and percolation threshold existed when the SiO_2_ content was around 20 wt %.

**Figure 9:**
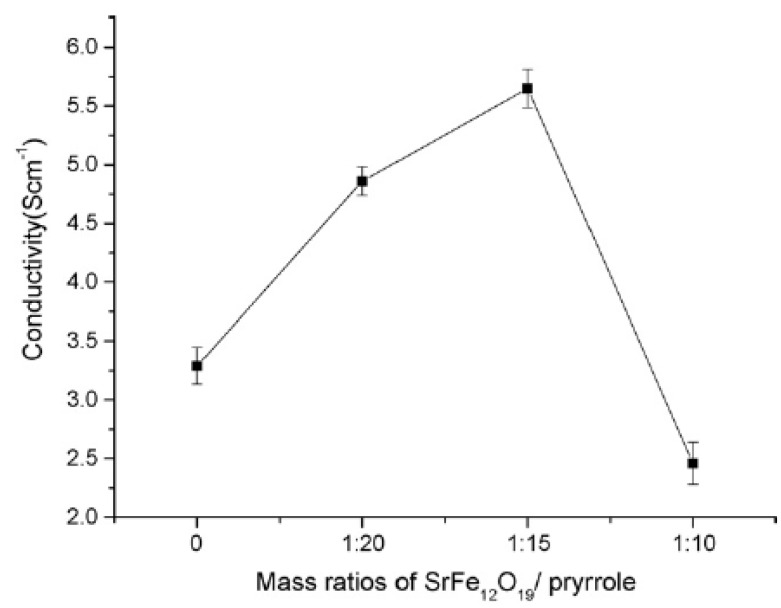
The conductivity variation curve of PPy/SrFe_12_O_19_ nanocomposite (adapted from [68]).

Zhang *et al.* synthesized PPy/nano-SrFe_12_O_19_ nanocomposites by *in situ* polymerization [69]. When the mass ratio of SrFe_12_O_19_ to pyrrole was less than 1:15, the conductivity (5.65 S/cm) of the PPy/SrFe_12_O_19_ nanocomposite was found to be higher than that of the pure PPy (3.29 S/cm) despite the insertion of the insulating SrFe_12_O_19_ particles (Figure 9).

Yu *et al.* manufactured hetrocyclically conjugated poly(3-hexylthiophene) (P3HT)/clay nanocomposites via *in situ* oxidative polymerization [70]. The polarization resistance of nanocomposites was increased from 10 to 1340 KΩ·cm^2^ with increasing content of clay nanoparticles to 10 wt %.

Ma *et al.* reported that polystyrene resin/ZnO nanocomposites were prepared by melt-blending [71]. The surface resistivity falls as the amount of ZnO increases. Adding 30 wt % of ZnO spherical particle and ZnO whisker reduced the surface resistivities of materials from 1.0 × 10^16^ to 8.98 × 10^12^ Ω/cm^2^ and to 9.57 × 10^10^ Ω/cm^2^, respectively. The amount of ZnO in polystyrene resin can be gradually increased to form a conductive network.

Xu *et al.* reported poly(ethylene glycol) (PEG) and poly(ethylene oxide) (PEO)/lithium intercalated tungsten disulfide (Li_x_WS_2_) nanocomposites [72]. They were prepared by exfoliation-adsorption technique. The resultant nanocomposites displayed pretty good conductivity in the range of 1 × 10^-2^ to 10^-3^ S/cm at ambient temperature. The conductivity likely originated from charge transfers.

Wang *et al.* prepared nanocomposite polymer electrolytes composed of poly(vinylidene fluoride (PVDF), lithium perchlorate (LiClO_4_) and TiO_2_ by a solution-cast method [73]. At a TiO_2_ content of 10 wt %, the solid and wet PVDF/LiClO_4_/TiO_2_ had the maximum conductivity of 7.1 × 10^-4^ and 1.8 × 10^-3^ S/cm, respectively. Pandey *et al.* also reported MnO/ion-conducting gel polymer electrolyte nanocomposite based on PVDF [74]. The optimum conductivity of the gel nanocomposite at 25 °C was ~8 × 10^-3^ S/cm at 3 wt % MgO. The temperature dependence of electrical conductivity was also reported: nanocomposites exhibited ionic conductivity of ~2 × 10^-3^ S/cm at 0 °C and 1 × 10^-3^ S/cm at 80 °C, respectively.

### 4.4. Optical properties

The optical properties of discontinuous metallic or granular composite films, consisting of metal nanoparticles embedded in a dielectric, have long been of interest [75]. Moreover, as the market of materials for optical applications expands, the need for novel materials with functionality and transparency increases. Polymer-based inorganic nanoparticle nanocomposites show great promise as they can provide the necessary stability and easy processability with interesting optical properties. As described earlier, metal nanoparticles show characteristic plasmon resonance modes during interaction with electromagnetic waves as a result of collective oscillations of free electrons and local enhancement of the electromagnetic field. This phenomenon largely depends on the particle size, shape, and the surrounding dielectric matrix. Particle plasmon resonances occur through absorption energies in the intra-band transitions and can be either dipolar excitation (one surface plasmon), in the case of spherical particles, or multipolar excitation of particles nonspherical in geometry [76].

The transparency of these composites depends upon the size and spatial distribution of inorganic particles in the polymer matrix. Pure polyamide and hybrid films containing various amounts of silica are transparent, the maximum transmittance was found in the hybrid film containing 5 wt % silica contents in the matrix, beyond which the transmittance was gradually decreased [54].

Chai *et al.* reported that PMMA/La_0.45_Ce_0.45_Tb_0.1_PO_4_ nanocomposite exhibited high transparency for visible light and showed strong green emission of Tb^3+^ (dominate at 543 nm) upon UV excitation [77]. Also, Wang *et al.* prepared nanocomposites from PMMA/SiO_2_ and PMMA/ZrO_2_ using a novel nonhydrolytic sol-gel process [59]. The transmittance of the nanocomposite films in the visible region remained above 95% even at 20 wt % inorganic content and was increased proportionally with decreasing inorganic content.

Avasthi *et al.* prepared PET/Ag nanocomposite that showed transmission in a narrow band around 320 nm [78]. With the increase in metal volume fraction, the transmission band becomes narrower and the attenuation of transmission intensity at 320 nm is increased because of superimposion of the absorption spectrum of the sputtered PET polymer and Ag nanostructure. The second feature in the UV-visible absorption spectra showed the broad band absorption. The extension from the visible region to IR region was attributed to the three-dimensional network of metal nanostructures.

Wu *et al.* reported that TiO_2_ nanoparticles prepared using sol-gel process were incorporated into the epoxy matrix by mechanical stirring [79]. Epoxy/TiO_2_ nanocomposite coating with refractive index of 1.668 can be obtained by adding 30 wt % the TiO_2_ nanoparticles into the epoxy resin. Also, all coating with different amounts of TiO_2_ exhibit excellent optical transparency of higher than 90%.

**Figure 10:**
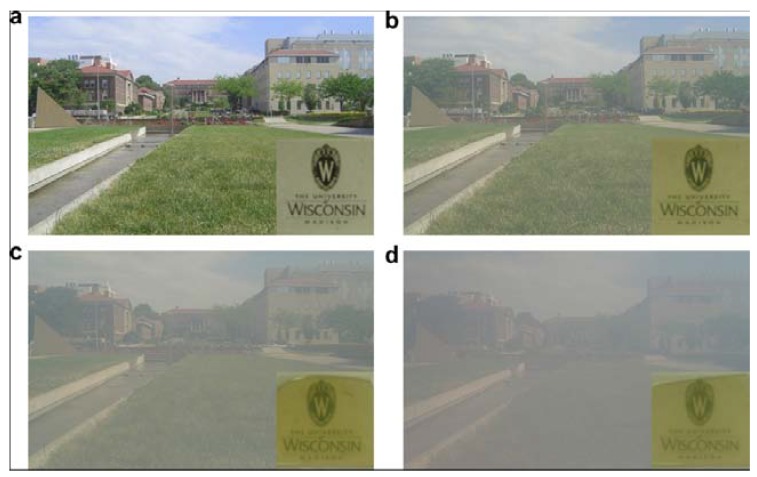
Transparencies of the (a) PC neat resin, (b) 1 wt % and (c) 2 wt % PC/alumina (SMA-coated), and (d) 2 wt % PC/alumina (untreated) (adapted from [7]).

Polycarbonate (PC)/alumina nanocomposites showed that the presence of the nanoparticles reduced the overall light transmittance of the nanocomposites [7]. The light transmittance was decreased by increasing the load of the nanoparticles. Furthermore, the poly(styrene-maleic anhydride copolymer (SMA)-coated alumina/PC nanocomposite had higher light transmittance than that of untreated alumina/PC nanocomposite (Figure 10).

### 4.5. Magnetic properties

Alam *et al.* prepared PANi/Fe_3_O_4_ nanocomposites that showed higher saturation magnetization of 3.2 emu/g at 300 K, revealing a super paramagnetic behavior [80]. Also, Qiaozhen *et al.* reported that PANi/Fe_3_O_4_@Au nanocomposites were fabricated by *in situ* polymerization and they showed higher saturation magnetization (M_S_) than the undoped PANi (27.35 × 10^-3^ emu/g) [81]. Upon decreasing the molar ratio of Au, the M_S_ of nanocomposites increased from 0.17 to 0.88 emu/g. The magnetic property of the nanocomposites is very close to surpramagnetic behavior.

**Figure 11:**
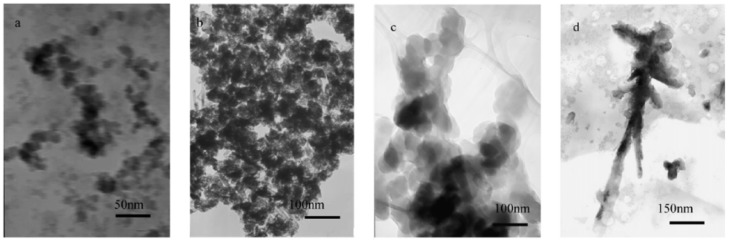
TEM of samples prepared with different pyrrole/SrFe_12_O_19_ mass ratio: (a) 0; (b) 10:1; (c) 15:1; (d) 20:1 (adapted from [69]).

Zhang *et al.* synthesized PPy/SrFe_12_O_19_ nancomposites via *in situ* polymerization [69]. PPy/nano-SrFe_12_O_19_ nancomposites were found with M_S_ and remnant magnetization (M_R_). It was found that the morphology of composites changed from sphere-like, conglobulation-like, and arborization-like structures with the increase of the pyrrole/SrFe_12_O_19_ mass ratio (Figure 11).

Xu *et al.* prepared poly(vinylidene difluoride) (PVDF)/Fe_3_O_4_ magnetic nanocomposite by a simple coprecipitation method [82]. The M_S_ and M_R_ of the PVDF/Fe_3_O_4_ nanocomposite increased with the increase of the Fe_3_O_4_ content. The M_S_ and M_R_ along the parallel direction were higher than those along the perpendicular direction at the same Fe_3_O_4_ content.

Zhan *et al.* showed polyimide (PI)/γ-Fe_2_O_3_ nancomposite films with superparamagnetic behavior [83]. With the increase of the Fe_3_O_4_ content from 2 wt % to 8 wt %, the M_S_ of PI/γ-Fe_2_O_3_ nanocomposite films increased from 1.354 × 10^-2^ A to 4.220 × 10^-2^ A. Therefore, the magnetic properties of nanocomposites can be adjusted by changing the Fe_3_O_4_ content.

**Figure 12:**
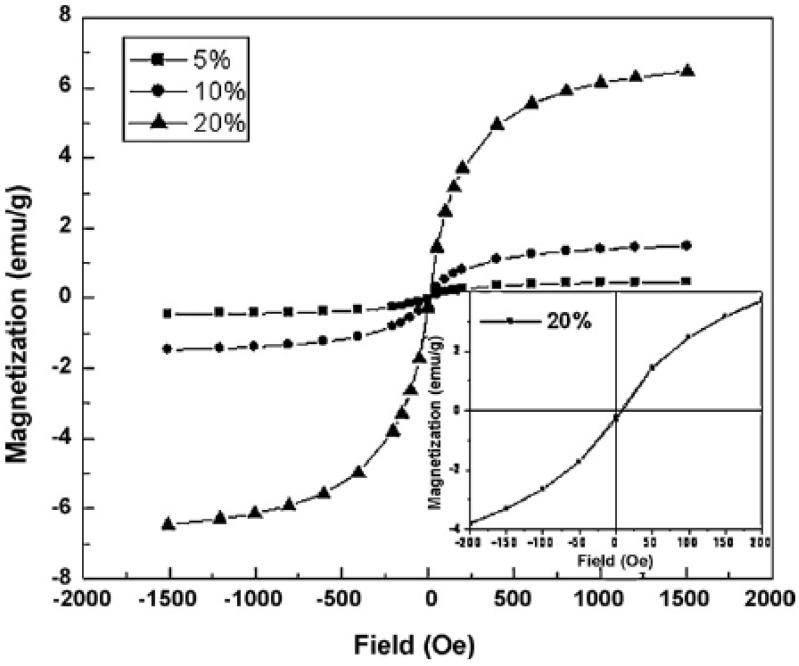
The magnetic hysteresis loops at 300 K for PEDOT/PSS-Fe_3_O_4_ nancomposites with different Fe_3_O_4_ content (adapted from Ref. [84]).

Sun *et al.* prepared poly(3,4-ethylenedioxythiophene/poly(styrene sulfonate)-Fe_3_O_4_ (PEDOT/PSS-Fe_3_O_4_) nancomposites [84]. The M_S_ was as high as 6.47 emu/g (20 wt % Fe_3_O_4_) at 300 K (Figure 12).

## 5. Applications of Nanocomposites

Table 3 presents potential applications of polymer-based inorganic particle nanocomposites [4,85]. The advantages of nanoscale inorganic particle incorporation into polymer matrices can lead to a number of applications that the incorporation of the analogous larger scale particles do not allow due to an insufficient property profile for utilization. These areas include barrier properties, membrane separation, UV screening, flame retardation, polymer blend compatibilization, electrical conductivity, impact modification, biomedical applications, *etc.* Hence, polymer-based inorganic nanoparticle nanocomposites emerging as new materials provide opportunities and rewards creating a new world of interest.

**Table 3:** Potential applications of polymer-based inorganic nanoparticle nancomposites (adapted from Refs. [4] and [85]).

Nanocomposites	Applications
Polycarprolactone/SiO_2_	Bone-bioerodible for skeletal tissue repair.
Polyimide/SiO_2_	Microelectronics.
PMMA/SiO_2_	Dental application, optical devices.
Polyethylacrylate/SiO_2_	Catalysis support, stationary phase for chromatography.
Poly(p-phenylene vinylene)/SiO_2_	Non-linear optical material for optical waveguides.
Poly(amide-imide)/TiO_2_	Composite membranes for gas separation.
Poly(3,4-ethylene-dioxythiphene)/ V_2_O_5_	Cathode materials for rechargeable lithium batteries.
Polycarbonate/SiO_2_	Abrasion resistant coating.
Shape memory polymers/SiC	Medical devices for gripping or releasing therapeutics within blood vessels.
Nylon-6/LS	Automotive timing-belt-TOYOTA.
Nylon-6/clay	Barrier films – Bayer AG
Nylon-6/clay	Films and bottles - Honeywell
Nylon-6, 12, 66/clay	Auto fuel systems - Ube
Nylon-6/PP/clay	Electrically conductive
UHMWPE/clay	Earthquake-resistance pipes – Yantai Haili Ind.& Commerce of China
Polypropylene/clay	Packaging - Clariant
PEO/LS	Airplane interiors, fuel tanks, components in electrical and electronic parts, brakes and tires.
PLA/LS	Lithium battery development.
PET/clay	Food packaging application. Specific examples include packaging for processed meats, cheese, confectionery, cereals and boil-in-the-bag foods, fruit juice and dairy products, beer and carbonated drinks bottles.
Thermoplastic olefin/clay	Beverage containers.
Polyimide/clay	Automotive step assists – GM Safari and Astra Vans.
Epoxy/MMT	Materials for electronics.
SPEEK/laponite	Direct methanol fuel cells.
EVA/clay	Wires and cables – Kabelwerk Eupen of Belgium
Unsaturated polyester/clay	Marine, transportation – Polymeric Supply

## 6. Conclusions

The hybridization of nanoparticles and polymers could improve various properties of resultant nanocomposites. The synergetic enhancements should originate from the specific attribute of each component. Toward an important objective for the development of multi-functional nanocomposites, is that the bulk physical properties should be easy to tailor for different purposes. Specifically, organic polymer-based nanocomposites generally have many advantages such as long-term stability and good processability, and inorganic nanoparticles possess outstanding optical, catalytic, electronic and magnetic properties. By combining the attractive functionalities of both components, resultant nanocomposites could potentially provide many applications in various areas such as automotive, aerospace, opto-electronics, *etc.*

## References

[1] Ajayan P.M., Schadler L.S., Braun P.V. (2003). Nanocomposite Science and Technology.

[2] Jordan J., Jacob K.I., Tannenbaum R., Sharaf M.A., Jasiuk I. (2005). Experimental trends in polymer nanocomposites - A review. Mater. Sci. Eng. A.

[3] Berta M., Lindsay C., Pans G., Camino G. (2006). Effect of chemical structure on combustion and thermal behaviour of polyurethane elastomer layered silicate nanocomposites. Polym. Degrad. Stabil..

[4] Sanchez C., Julián B., Belleville P., Popall M. (2005). Applications of hybrid organic-inorganic nanocomposites. J. Mater. Chem..

[5] Hiemenz P., Rajagopalan R. (1997). Principles of Colloid and Surface Chemistry.

[6] Suryanarayana C., Froes F.H. (1992). The structure and mechanical properties of metallic nanocrystals. Metall. Trans. A.

[7] Chandra A., Turng L.S., Gopalan P., Rowell R.M., Gong S. (2008). Study of utilizing thin polymer surface coating on the nanoparticles for melt compounding of polycarbonate/alumina nanocomposites and their optical properties. Comp. Sci. Technol..

[8] Osman M.A., Rupp J.E.P., Suter U.W. (2005). Effect of non-ionic surfactants on the exfoliation and properties of polyethylene-layered silicate nanocomposites. Polymer.

[9] Cho J.W., Paul D.R. (2001). Nylon 6 nanocomposites by melt compounding. Polymer.

[10] Chang J.H., An Y.U., Cho D., Giannelis E.P. (2003). Poly(lactic acid) nanocomposites: comparison of their properties with montmorillonite and synthetic mica (II). Polymer.

[11] Paul D.R., Robeson L.M. (2008). Polymer nanotechnology: nanocomposites. Polymer.

[12] Yasmin A., Luo J.J., Abot J.L., Daniel I.M. (2006). Mechanical and thermal behavior of clay/epoxy nanocomposites. Comp. Sci. Technol..

[13] Wang K., Chen L., Wu J., Toh M.L., He C., Yee A.F. (2005). Epoxy nanocomposites with highly exfoliated clay: Mechanical properties and fracture mechanisms. Macromolecules.

[14] Lippens P.E., Lannoo M. (1989). Calculation of the band gap for small CdS and ZnS crystallites. Phys. Rev. B.

[15] Palik E., Ghosh G. (1985). Handbook of Optical Constants of Solids.

[16] Prevenslik T.V. (2000). Acoustoluminescence and sonoluminescence. J. Lumin..

[17] Hiemenz P., Rajagopalan R. (1997). Principles of Colloid and Surface Science.

[18] Wu T., Ke Y. (2007). Melting, crystallization and optical behaviors of poly (ethylene terephthalate)-silica/polystyrene nanocomposite films. Thin Solid Films.

[19] Shenhar R., Norsten T.B., Rotello V.M. (2005). Polymer-mediated nanoparticle assembly: structural control and applications. Adv. Mater..

[20] Han K., Yu M. (2006). Study of the preparation and properties of UV-blocking fabrics of a PET/TiO_2_ nanocomposite prepared by *in situ* polycondensation. J. Appl. Polym. Sci..

[21] Guo Z., Pereira T., Choi O., Wang Y., Hahn H.T. (2006). Surface functionalized alumina nanoparticle filled polymeric nanocomposites with enhanced mechanical properties. J. Mater. Chem..

[22] Cushing B., Kolesnichenko V., O'Connor C. (2004). Recent advances in the liquid-phase syntheses of inorganic nanoparticles. Chem. Rev..

[23] Alexandre M., Dubois P. (2000). Polymer-layered silicate nanocomposites: Preparation, properties and uses of a new class of materials. Mater. Sci. Eng. R..

[24] Ogata N., Kawakage S., Ogihara T. (1997). Structure and thermal/mechanical properties of poly(ethylene oxide)-clay mineral blends. Polymer.

[25] Jeon H.G., Jung H.T., Lee S.W., Hudson S.D. (1998). Morphology of polymer/silicate nanocomposites: high density polyethylene and a nitrile copolymer. Polym. Bull..

[26] Okamoto M., Morita S., Kim Y.H., Kotaka T., Tateyama H. (2000). Synthesis and structure of smectic clay/poly(methyl methacrylate) and clay/polystyrene nanocomposites via *in situ* intercalative polymerization. Polymer.

[27] Okamoto M., Morita S., Kotaka T. (2001). Dispersed structure and ionic conductivity of smectic clay/polymer nanocomposites. Polymer.

[28] Yao K.J., Song M., Hourston D.J., Luo D.Z. (2002). Polymer/layered clay nanocomposites 2: polyurethane nanocomposites. Polymer.

[29] Vaia R.A., Giannelis E.P. (1997). Lattice model of polymer melt intercalation in organically-modified layered silicates. Macromolecules.

[30] Kawasumi M., Hasegawa N., Kato M., Usuki A., Okada A. (1997). Preparation and mechanical properties of polypropylene-clay hybrids. Macromolecules.

[31] Gyoo P., Venkataramani S., Kim S. (2006). Morphology, thermal, and mechanical properties of polyamide 66/clay nanocomposites with epoxy-modified organoclay. J. Appl. Polym. Sci..

[32] Erdem N., Cireli A., Erdogan U. (2009). Flame retardancy behaviors and structural properties of polypropylene/nano-SiO_2_ composite textile filaments. J. Appl. Polym. Sci..

[33] Du H., Xu G., Chin W., Huang L., Ji W. (2002). Synthesis, characterization, and nonlinear optical properties of hybridized CdS-polystyrene nanocomposites. Chem. Mater..

[34] Carrado K.A., Xu L. (1998). *In situ* synthesis of polymer-clay nanocomposites from silicate gels. Chem. Mater..

[35] Tomasko D.L., Han X., Liu D., Gao W. (2003). Supercritical fluid applications in polymer nanocomposites. Curr. Opin. Solid St. Mater. Sci..

[36] Park S.S., Bernet N., De La Roche S., Hahn H.T. (2003). Processing of iron oxide-epoxy vinyl ester nanocomposites. J. Comp. Mater..

[37] Evora V.M.F., Shukla A. (2003). Fabrication, characterization, and dynamic behavior of polyester/TiO_2_ nanocomposites. Mater. Sci. Eng. A.

[38] Aymonier C., Bortzmeyer D., Thomann R., Mülhaupt R. (2003). Poly(methyl methacrylate)/palladium nanocomposites: synthesis and characterization of the morphological, thermomechanical, and thermal properties. Chem. Mater..

[39] Avadhani C.V., Chujo Y. (1997). Polyimide-silica gel hybrids containing metal salts: preparation via the sol-gel reaction. Appl. Organometal. Chem..

[40] Liu J., Gao Y., Wang F., Li D., Xu J. (2002). Preparation and characteristic of a new class of silica/polyimide nanocomposites. J. Mater. Sci..

[41] Kickelbick G. (2003). Concepts for the incorporation of inorganic building blocks into organic polymers on a nanoscale. Prog. Polym. Sci..

[42] Wang K., Choi M., Koo C., Choi Y., Chung I. (2001). Synthesis and characterization of maleated polyethylene/clay nanocomposites. Polymer.

[43] Chaichana E., Jongsomjit B., Praserthdam P. (2007). Effect of nano-SiO2 particle size on the formation of LLDPE/SiO2 nanocomposite synthesized via the *in situ* polymerization with metallocene catalyst. Chem. Eng. Sci..

[44] Fornes T., Yoon P., Keskkula H., Paul D. (2001). Nylon 6 nanocomposites: the effect of matrix molecular weight. Polymer.

[45] Homminga D., Goderis B., Mathot V., Groeninckx G. (2006). Crystallization behavior of polymer/montmorillonite nanocomposites. Part III. Polyamide-6/montmorillonite nanocomposites, influence of matrix molecular weight, and of montmorillonite type and concentration. Polymer.

[46] Zhao Q., Samulski E. (2005). *In situ* polymerization of poly (methyl methacrylate)/clay nanocomposites in supercritical carbon dioxide. Macromolecules.

[47] Zerda A., Caskey T., Lesser A. (2003). Highly concentrated, intercalated silicate nanocomposites: synthesis and characterization. Macromolecules.

[48] Pattanayak A., Jana S.C. (2005). Properties of bulk-polymerized thermoplastic polyurethane nanocomposites. Polymer.

[49] Min K.D., Kim M.Y., Choi K.Y., Lee J.H., Lee S.G. (2006). Effect of layered silicates on the crystallinity and mechanical properties of HDPE/MMT nanocomposite blown films. Polym. Bull..

[50] Lee H., Lin L. (2006). Waterborne polyurethane/clay nanocomposites: novel effects of the clay and its interlayer ions on the morphology and physical and electrical properties. Macromolecules.

[51] Fornes T.D., Paul D.R. (2003). Crystallization behavior of nylon 6 nanocomposites. Polymer.

[52] Zhang X., Simon L.C. (2005). *In situ* polymerization of hybrid polyethylene-alumina nanocomposites. Macromol. Mater. Eng..

[53] Chae D.W., Kim B.C. (2005). Characterization on polystyrene/zinc oxide nanocomposites prepared from solution mixing. Polym. Adv. Technol..

[54] Sarwar M.I., Zulfiqar S., Ahmad Z. (2008). Polyamide-silica nanocomposites: Mechanical, morphological and thermomechanical investigations. Polym. Int..

[55] Lu X., Lu N., Gao J., Jin X., Lu C. (2007). Synthesis and properties of ZnS polyimide nanocomposite films. Polym. Int..

[56] Reddy C.S., Das C.K. (2006). Polypropylene-nanosilica-filled composites: effects of epoxy-resin-grafted nanosilica on the structural, thermal, and dynamic mechanical properties. J. Appl. Polym. Sci..

[57] Yu S., Hing P., Hu X. (2002). Thermal conductivity of polystyrene-aluminum nitride composite. Compos. Part A.

[58] Lee G., Park M., Kim J., Lee J., Yoon H. (2006). Enhanced thermal conductivity of polymer composites filled with hybrid filler. Compos. Part A.

[59] Wang H., Xu P., Zhong W., Shen L., Du Q. (2005). Transparent poly(methyl methacrylate)/silica/zirconia nanocomposites with excellent thermal stabilities. Polym. Degrad. Stabil..

[60] Du L., Qu B., Zhang M. (2007). Thermal properties and combustion characterization of nylon 6/MgAl-LDH nanocomposites via organic modification and melt intercalation. Polym. Degrad. Stabil..

[61] Du L., Qu B. (2006). Structural characterization and thermal oxidation properties of LLDPE/MgAl-LDH nanocomposites. J. Mater. Chem..

[62] Lu H., Hu Y., Li M., Chen Z., Fan W. (2006). Structure characteristics and thermal properties of silane-grafted-polyethylene/clay nanocomposite prepared by reactive extrusion. Comp. Sci. Technol..

[63] Qiu L., Chen W., Qu B. (2005). Structural characterisation and thermal properties of exfoliated polystyrene/ZnAl layered double hydroxide nanocomposites prepared via solution intercalation. Polym. Degrad. Stabil..

[64] Valera-Zaragoza M., Ramírez-Vargas E., Medellín-Rodríguez F.J., Huerta-Martínez B.M. (2006). Thermal stability and flammability properties of heterophasic PP-EP/EVA/organoclay nanocomposites. Polym. Degrad. Stabil..

[65] Su S.J., Kuramoto N. (2000). Processable polyaniline-titanium dioxide nanocomposites: effect of titanium dioxide on the conductivity. Synthet. Metal..

[66] Mo T.C., Wang H.W., Chen S.Y., Yeh Y.C. (2008). Synthesis and dielectric properties of polyaniline/titanium dioxide nanocomposites. Ceram. Int..

[67] Olad A., Rashidzadeh A. (2008). Preparation and anticorrosive properties of PANI/Na-MMT and PANI/O-MMT nanocomposites. Prog. Org. Coat..

[68] Tang Q., Sun X., Li Q., Lin J., Wu J. (2009). Preparation and electrical conductivity of SiO_2_/polypyrrole nanocomposite. J. Mater. Sci..

[69] Zhang C., Li Q., Ye Y. (2009). Preparation and characterization of polypyrrole/nano-SrFe_12_O_19_ composites by *in situ* polymerization method. Synthet. Metal..

[70] Yu Y.H., Jen C.C., Huang H.Y., Wu P.C., Huang C.C., Yeh J.M. (2004). Preparation and properties of heterocyclically conjugated poly(3-hexylthiophene)-clay nanocomposite materials. J. Appl. Polym. Sci..

[71] Ma C.C.M., Chen Y.J., Kuan H.C. (2006). Polystyrene nanocomposite materials - preparation, mechanical, electrical and thermal properties, and morphology. J. Appl. Polym. Sci..

[72] Xu B.H., Lin B.Z., Sun D.Y., Ding C. (2007). Preparation and electrical conductivity of polyethers/WS_2_ layered nanocomposites. Electrochim. Acta.

[73] Wang Y.J., Kim D. (2007). Crystallinity, morphology, mechanical properties and conductivity study of *in situ* formed PVdF/LiClO_4_/TiO_2_ nanocomposite polymer electrolytes. Electrochim. Acta.

[74] Pandey G.P., Agrawal R.C., Hashmi S.A. (2009). Magnesium ion-conducting gel polymer electrolytes dispersed with nanosized magnesium oxide. J. Power Sources.

[75] Garnett J. (1906). Colours in metal glasses, in metallic films, and in metallic solutions. II. Philos. Trans. R. Soc. London A.

[76] Biswas A., Aktas O.C., Kanzow J., Saeed U., Strunskus T., Zaporojtchenko V., Faupel F. (2004). Polymer-metal optical nanocomposites with tunable particle plasmon resonance prepared by vapor phase co-deposition. Mater. Lett..

[77] Chai R., Lian H., Yang P., Fan Y., Hou Z., Kang X., Lin J. (2009). *In situ* preparation and luminescent properties of LaPO_4_:Ce^3+^, Tb^3+^ nanoparticles and transparent LaPO_4_:Ce^3+^, Tb^3+^/PMMA nanocomposite. J. Colloid. Interf. Sci..

[78] Avasthi D.K., Mishra Y.K., Kabiraj D., Lalla N.P., Pivin J.C. (2007). Synthesis of metal-polymer nanocomposite for optical applications. Nanotechnology.

[79] Chau J.L.H., Tung C.T., Lin Y.M., Li A.K. (2008). Preparation and optical properties of titania/epoxy nanocomposite coatings. Mater. Lett..

[80] Alam J., Riaz U., Ahmad S. (2007). Effect of ferrofluid concentration on electrical and magnetic properties of the Fe_3_O_4_/PANI nanocomposites. J. Magn. Magn. Mater..

[81] Yu Q., Shi M., Cheng Y., Wang M., Chen H. (2008). Fe3O4@ Au/polyaniline multifunctional nanocomposites: their preparation and optical, electrical and magnetic properties. Nanotechnology.

[82] Xu C., Ouyang C., Jia R., Li Y., Wang X. (2009). Magnetic and optical properties of poly(vinylidene difluoride)/Fe_3_O_4_ nanocomposite prepared by coprecipitation approach. J. Appl. Polym. Sci..

[83] Zhan J., Tian G., Jiang L., Wu Z., Wu D., Yang X., Jin R. (2008). Superparamagnetic polyimide/γ-Fe_2_O_3_ nanocomposite films: preparation and characterization. Thin Solid Films.

[84] Sun D.C., Sun D.S. (2009). The synthesis and characterization of electrical and magnetic nanocomposite: PEDOT/PSS-Fe_3_O_4_. Mater. Chem. Phys..

[85] Camargo P., Satyanarayana K., Wypych F. (2009). Nanocomposites: synthesis, structure, properties and new application opportunities. Mater. Res..

